# An experimental determination of the drag coefficient of a Mens 8+ racing shell

**DOI:** 10.1186/2193-1801-3-512

**Published:** 2014-09-10

**Authors:** James G Buckmann, Samuel D Harris

**Affiliations:** Spencer Laboratory, University of Delaware, Newark, DE 19711 USA

**Keywords:** Racing, Shell, Rowing, Crew, Boat, Hydrodynamic, Aerodynamic, Drag, Force

## Abstract

This study centered around an experimental analysis of a Mens Lightweight Eight racing shell and, specifically, determining an approximation for the drag coefficient. A testing procedure was employed that used a Global Positioning System (GPS) unit in order to determine the acceleration and drag force on the shell, and through calculations yield a drag coefficient. The testing was run over several days in numerous conditions, and a 95% confidence interval was established to capture the results. The results obtained, over these varying trials, maintained a successful level of consistency. The significance of this study transcends the determination an approximation for the drag coefficient of the racing shell; it defined a successful means of quantifying performance of the shell itself. The testing procedures outlined in the study represent a uniform means of evaluating the factors that influence drag on the shell, and thus influence speed.

## Introduction

In the sport of rowing, scientific research on racing shells is limited due to the relatively small community and the lack of funding. Much of the knowledge regarding hydro craft of such a nature surrounds Yachts and the Americas Cup. Some of this theory can be seen in the Theoretical Background. While certain aspects of these basic naval architecture principles loosely apply to a racing shell, they do not explicitly define the mechanics of the shell. That the racing shell is a displacement hull, that is, it is supported primarily through buoyancy rather than lift, is its main deviation from standard yacht designs. Because of this principle, the sailboat with the design most similar to the racing shell is the catamaran, both of which have long, thin hull shapes.

Because of the principle differences between a racing shell and standard planing hydrocraft, specifics regarding the dynamics of a racing shell are frequently disputed. Though there has been research conducted on racing shells in the past, the knowledge base is rather limited. There are a number of studies on the biomechanics of the rowing motion, but few that deal directly with the shell design (Baudouin & Hawkins 2010), (Day et al. 2011a), (Day et al. 2011b), (Serveto et al. 2009). The aims and results of these studies are taken into consideration in this project. Of these studies, it is noted that none included an experimental analysis of a large shell, presumably because of the lack of availability of eight-man boats (known as an 8+). It is with great fortune that the University of Delaware Men’s Crew Team was able to be used as a resource, as data could be obtained on 8+ racing shells. It is noted that no other public data was found on eights. Previous research, supposedly conclusive, has been conducted on large shells for the US National Team, though the details of this study have not been made public. It is the lack of prior research that gives this study value.

The aim of this study is to define a method of testing a racing shell to determine a universal drag coefficient. That is, the testing procedure outlined in this study can be run on a shell to obtain a preliminary drag coefficient, at which point a design change would be made to the shell, and the test would be re-run to identify how the drag coefficient was changed.

While certain resources, such as shell and rower availability, were commodities, funding, time and environmental conditions were not. As such, the drag coefficient defined in this study is limited to an experimental approximation.

It is noted that the drag coefficient defined in this study does not seek to isolate hydrodynamic and aerodynamic drag. This is a limitation that was implemented so as to avoid the requirement to include theoretical approximations and maintain purely experimental results. Similarly, the effect of wind and current on the drag coefficient could be analyzed as well.

## Theoretical background

In naval architecture and ship design there are a number of design ratios that can be applied to relate speed with certain shell properties such as length, area and displacement. The speed length ratio, SLR, specifically is the ratio of maximum speed, *v*, to the square root of the water line length, LWL (Sponberg 2010).


Equation 1 – Speed-Length Ratio

This ratio applies to craft that transition from displacement to planing hulls, and is not directly applicable to a racing shell. A hull with a SLR of around 1.3 is in displacement mode, 1.3-2.5 is semi-planing, and 2.5 and above is fully planing (Sponberg 2010). If it is assumed that a racing shell applies to this principle and has a SLR of 1.3, it can be said that as water line length increases, maximum potential speed increases as well. This would be an answer to the frequently disputed question of how shell length affects speed. However, as stated prior, these naval architecture principles are not reliable for long, thin, displacement hulls.

The prismatic coefficient, C_p_, is the ratio of the volume of displaced water to the maximum area times the length of the water line. When the speed length ratio applies, the volume of a hull can be designed under guidance of the C_p_ to support the shells speed at a corresponding speed length ratio. With regards to long, thin displacement hulls, the prismatic coefficient tends to approach 0.7. This value can loosely be used to relate under water volume of a shell to the length and maximum beam area.

Since these largely accepted ratios for planing hydro craft do not strictly apply to racing shells, it is important to define the dynamics that impact the shell. This theory is used as the basis of the experimental analysis.

With regards to the kinetics of a shell, three categories of retarding hydrodynamic forces act on it; form drag, skin friction, and wave drag. Form drag is a force proportional to the square of the boat speed that depends on a drag coefficient which incorporates area of the shell and density of water.

Skin friction is based on the friction between the water and the shell. A thin film of water is accelerated to the speed of the shell, creating a boundary layer. Based on principles of the flow of water over a flat plate, skin friction on the shell is approximately proportional to the shell velocity to the 1.5^th^ power and varies based on water depth. Wave drag is the drag force on the shell caused by creation of waves. Since a racing shell is a long thin craft, wave drag is not a significant source of drag.

Aerodynamic drag acts in a similar manner but holds less of an impact on the total drag as the density of air is much less than that of water.

Because of these characteristics and the fact that at greater speeds form drag is the most influential drag, as can be seen by the relationship between drag force and velocity for the different modes of drag, the equation for form drag is used as the baseline to determine the drag coefficient. It is noted that this is a significant assumption necessary for the goals of this study.

This study defined a universal drag coefficient that includes both aerodynamic and hydrodynamic effects. The drag coefficient, C_D_, is defined by the drag force, F_D_, and the shell velocity (*v*) as shown in Equation 2 (Kleshnev 2010).


Equation 2 – Definition of Drag Coefficient

Regarding the theoretical analysis, a general knowledge of the relationship between speed and drag force was required. Based on the information and equations provided above, it is seen that the drag force could be defined as a constant times velocity raised to a power between 1.5 and 2 (White 2011). The constant in these derived equations approximately represents the above defined drag coefficient.

Using the theoretical principles stated above, a value for the coefficient of drag was determined. The following section describes the experimental process that this study employs.

## Testing methods and procedures

The equipment required for this study is one 10 Hz GPS Logger and supporting software, one racing shell with appropriate oarsmen, coxswain and oars, and two scales for measuring mass of the participants and equipment. On this occasion, a Resolute Lightweight 8+ was used with Concept 2 oars with a standard vortex blade. The rowers averaged 165 lbs (74.8 kg) in weight and the coxswain was 125 lbs (56.7 kg). The mass of the oars was determined by putting them on a scale accurate to 0.2 lbs and the mass of the shell was measured by placing it on two scales and summing the read value. Table 1 below shows these mass constants that were used in determining the drag coefficient.

**Table 1:** **Drag component masses**

Component	Mass (kg)
Rowers	655
Shell	89
Oars	22.5

A QStarz 10 Hz Global Positioning System (GPS) unit (Qstarz Bt-Q100ex with differential GPS support) was secured to the shell and used to determine the velocity of the system during the testing process. The GPS acquisition rate of ten points per second provided sufficient precision for this study.

When developing a means of testing so as to approximate the drag coefficient, the repulsive force on the shell by the water was isolated.

Over the course of a stroke, there are a multitude of forces acting on the shell. While the oars are in the water and the rowers are driving, propulsive forces act on the system. Whenever the rowers are moving up or down the tracks, there are reactive forces acting on the shell, either because the rowers are drawing themselves up the slide, or pushing against the footplate. Because of the multitude of acting forces, a specific methodology of testing was employed.

Prior to execution of the testing, coaches, coxswains and oarsmen were briefed on the study, its intents, and what was required of them. Adequate warm-up was allowed before the testing took place. The 8+ took 15 or 20 strokes at full pressure and racing speed and then immediately stopped. The rowers’ movements when they stop rowing were vital. Each blade was feathered and did not drag on the water and the boat was balanced. This ensured that the only hydrodynamic force acting on the system was the repulsive force, not an induced drag from the blades on the water. Feathering the blades minimized the aerodynamic drag. This position was held for a timeframe of approximately 10 seconds so as to obtain as much data in the deceleration phase as possible. Once completed, the rowers were instructed to “sit easy”.

Once the trial was completed, the data from the GPS was exported to a simple CSV format spreadsheet using the accompanying software and each trial was manually extracted. During the deceleration phase for each trial, a Velocity vs. Time graph was produced. This curve was derived to determine acceleration, which lead to the repulsive force. This was the drag force used to calculate the drag coefficient. It is noted that the maximum acceleration (*a*) was used since this will most closely resemble the true drag force on the shell at full pressure over the course of the entire stroke. The corresponding velocity at this acceleration was similarly used for the calculations. The mass of the system (*m*) was calculated by summing the mass of the individual components.


Equation 3 – Drag Coefficient Calculation

## Results and Analysis

**Figure 1:**
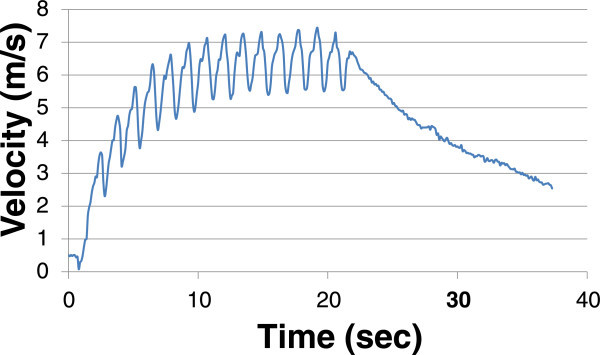
**V vs. T: entire trial.**

**Figure 2:**
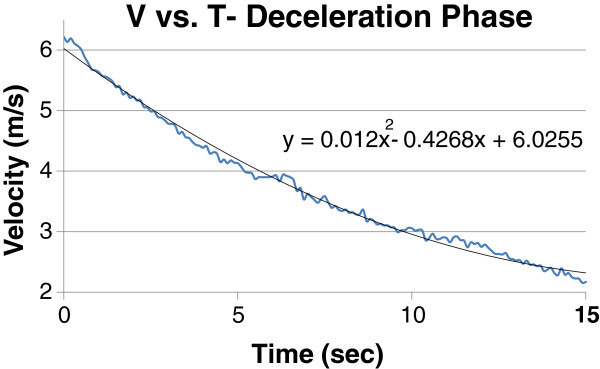
**Isolated velocity deceleration curve with trendline.**

The graph below, in Figure 1, shows the velocity curve during one trial run, including both the strokes to build the pressure and the pure deceleration phase. It can be seen that, while the curve holds smooth in general, there were a number of outliers. These discrepancies are more pronounced in Figure 2. The explanation for these inconsistencies can be anything from jitter of the GPS unit to the shell hitting an unexpected wave or a gust of wind. For practicality and averaging purposes, these outliers were manually be removed from the data set in order to obtain the desired accuracy, defined by R^2^ values approaching 1.

From the above trial, the velocity curve from the deceleration phase was isolated and the outliers were removed. This velocity curve, along with its corresponding trend line, is shown in Figure 2. A second order polynomial for the velocity curve was used to fit the shape during the deceleration phase.

It is noted that this second order polynomial does not seek to explicitly define the behavior of the curve. Its purpose is to quantitatively assign a trendline to the velocity curve during the deceleration phase and allow for an acceleration to be derived in order to determine force.

The instant when the rowers stopped moving and the shell was in its pure deceleration phase is defined as the initial condition *t* = 0.

**Figure 3:**
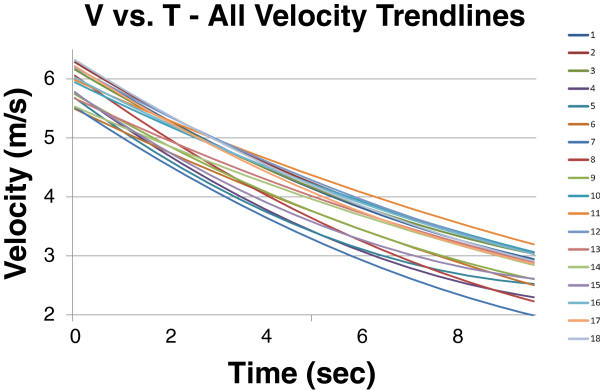
**All overlaid velocity trendlines.**

Velocity trendlines obtained from all applicable trials are shown in Figure 3. The curves showed that the general deceleration was similar between all trials, though speed itself varied on the order of 1 m/s. Though velocities were adjusted in each trial using an approximation to account for current, this variability could stem from potential remaining discrepancies.

With the velocity, deceleration trendlines derived for acceleration, *a*(0) and *v*(0) were calculated as shown in Table 2, and the instantaneous drag coefficient at time t = 0 was determined using the methodology stated in Equation 3. Table 2 shows the acceleration, velocity and calculated drag coefficient for each trial. The drag force equations for each corresponding drag coefficient (F(*v*)_1_), as determined by the principles outlined in Equation 3, are shown in Table 2 as well.

**Table 2:** **Drag Coefficient Results**

Trial	***a***(0)	***v***(0)	C _d_	F(v) _1_	F(v) _2_	Conditions*
1	0.5009	5.969	9.94	F = 9.94*v* ^2^	F = 20.6*v* ^1.6^	~0 m/s t.c.,
						5 mph h.w.
2	0.4939	6.041	9.59	F = 9.59*v* ^2^	F = 14.7*v* ^1.9^	~0 m/s t.c.,
						5 mph h.w.
3	0.4861	5.921	9.82	F = 9.82*v* ^2^	F = 13.6*v* ^1.9^	~0 m/s t.c.,
						5 mph h.w.
4	0.5956	5.485	13.7	F = 9.13.7*v* ^2^	F = 14.8*v* ^1.9^	0.5 m/s h.c.,
						10 mph h.w.
5	0.5933	5.388	14.1	F = 14.1*v* ^2^	F = 18.4*v* ^2.0^	0.5 m/s h.c.,
						10 mph h.w.
6	0.3910	5.298	9.94	F = 9.94*v* ^2^	F = 22.0*v* ^1.7^	0.25 m/s h.c.,
						15 mph h.w.
7	0.5399	5.258	13.6	F = 13.6*v* ^2^	F = 30.7*v* ^1.8^	0.25 m/s h.c.,
						15 mph h.w.
8	0.5831	5.771	12.2	F = 12.2*v* ^2^	F = 17.8*v* ^2.0^	0.25 m/s h.c.,
						15 mph h.w.
9	0.4775	5.507	11.1	F = 11.1*v* ^2^	F = 18.1*v* ^1.8^	0.25 m/s h.c.,
						15 mph h.w.
10	0.3986	5.748	8.64	F = 8.64	F = 7.3*v* ^2.1^	0.5 m/s t.c.,
						10 mph t.w.
11	0.3643	5.803	7.80	F = 7.80*v* ^2^	F = 18.9*v* ^1.4^	0.5 m/s t.c.,
						10 mph t.w.
12	0.4462	5.976	8.91	F = 8.91*v* ^2^	F = 8.5*v* ^2.1^	0.5 m/s t.c.,
						10 mph t.w.
13	0.3824	5.482	9.12	F = 9.12*v* ^2^	F = 12.9*v* ^1.8^	0.5 m/s t.c.,
						10 mph t.w.
14	0.3558	5.356	8.91	F = 8.91*v* ^2^	F = 13.5*v* ^1.7^	~0 m/s t.c.,
						5 mph h.w.
15	0.5630	5.494	12.9	F = 12.9*v* ^2^	F = 9.7*v* ^2.1^	~0 m/s t.c.,
						5 mph h.w.
16	0.4268	6.0255	9.01	F = 9.01*v* ^2^	F = 9.1*v* ^2.0^	~0 m/s t.c.,
						5 mph h.w.
17	0.5190	5.964	10.3	F = 10.3*v* ^2^	F = 8.3*v* ^2.1^	~0 m/s t.c.,
						5 mph h.w.
18	0.5087	6.074	9.75	F = 9.75*v* ^2^	F = 9.5*v* ^2.0^	~0 m/s t.c.,
						5 mph h.w.

*t.c. indicates tail current.

h.c. indicates head current.

t.w. indicates tail wind.

h.w. indicates head wind.

The above dataset yielded an average drag coefficient of 10.5 and a standard deviation of 1.9. The 95% confidence interval for the data, when using acceleration and velocity at this point, was between 9.6 and 11.4.

**Figure 4:**
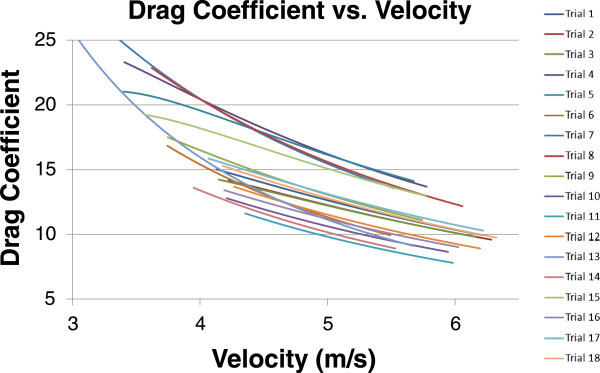
**Drag coefficient variance with velocity.**



The behavior of the Drag Coefficient was further investigated by analyzing how it varies with velocity. Equation 4 is a variation on Equation 3 which shows how C_D_ varies with time. The results from this equation can then be plotted against velocity in order to determine how the defined C_D_ varies with the speed of the shell. This plot is shown in Figure 4 and is interpreted in the Conclusions section.

Equation 4 – Drag Coefficient Variance with Time

As a means of verifying this data and to analyze impacts of wind and current conditions, Drag Force vs. Velocity graphs were generated for each trial.

**Figure 5:**
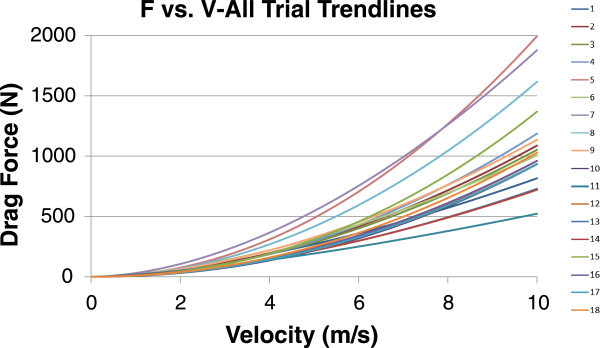
**Drag force vs. V trendlines.**

The velocity and acceleration equations, derived using the trendlines shown in Figures 2 and 3, were used in conjunction with the known masses outlined in the Procedures section to obtain the Drag vs. V trendlines shown in Figure 5. Table 2 outlines the Drag Force equations for each of these trendlines (F(*v*)_2_) and comments on the conditions, both wind and water, for each trial. The impact and significance of these conditions is discussed further in the Conclusions section.

**Figure 6:**
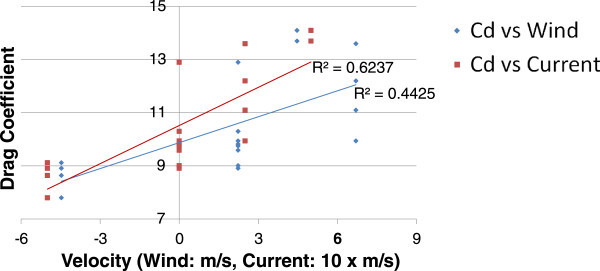
**Drag coefficient vs. conditions.**

Using the drag coefficients and conditions identified in Table 2, a plot was made that compares the condition with the drag coefficient for each trial. Figure 6 contains these results and separates effect of current, red points and trendline, with effect of wind, blue points and trendline. Positive wind and current velocities indicate head-conditions (headwind, head-current) while negative indicates tail-conditions. It can be seen that there is a loose correlation in the dataset indicating that as head-condition velocity increases, the drag coefficient increases as well. Similarly, from the few trials that contained tail-conditions, the drag coefficient is calculated to be less than that of those with head-conditions. This general conclusion is the expected result. The original testing procedure was not designed to include this analysis and the conditions used were approximations. It is expected that the results would be more precise and conclusive if other equipment, such as an anemometer, was incorporated into the study. As such, and due to the positive results of this brief analysis, it is desired that a new testing procedure be developed with the specific intent of determining the relationship between drag coefficient and present conditions.

## Conclusions

These results provide an initial baseline set of data. However, as noted prior, time and conditions were not ideal, so as the number of trials increases, more specifically if the number of trials increase on one day or in one specific set of conditions, precision of the study is expected to increase drastically.

Since testing was spread out over a number of different days and conditions, variability in the results was expected. However, the confidence interval determined was more accurate than expected. Similarly, when viewing trials of one day, the data seems to be appropriately more precise. For example, Trials 1 – 3 were all conducted on the same day in the same conditions. The drag coefficients determined on this day ranged from 9.59 to 9.94 over three trials.

This statement was backed up when viewing the results shown in Table 2. Notable C_D_ outliers as seen in Trials 4, 5, 7, 8 and 11 are shown to have occurred in testing with much more severe conditions, both wind and current, than those of Trials 1 – 3. This proves the credibility of the testing methodology and encourages more results to be taken over one day and one condition.

Table 2 includes two different Force vs. Velocity equations, one using the F = C_D_*v*^2^ format and one using the F = C_D_*v*^A^ format where A is a constant depending on each trial. The first equation is derived from calculating the Drag Coefficient based on the methods outlined in the Procedures section while the second equation is from the Force vs. Velocity trendlines shown in Figure 5. It is noted that the second equation produced less precise R^2^ values than the first.

With regards to how the drag coefficient varies with velocity, each trial exhibited similar behavior and, when compared with conventional Drag Coefficient vs. Velocity or Reynolds Number charts for various objects, the results found in this study contain similarities in terms of shape and characteristics. That being said, this study did not explicitly seek to define the Drag Coefficient vs. Velocity relationship. It is greatly desired to run further, more specific testing, to verify the behavior of this relationship. Regarding outlying trials for the C_D_ vs. V chart, similar outliers are seen in this chart as noted above. Trials 4, 5, 7, 8 and 13 all depicted greater drag coefficients than the cluster.

With the above information, it can be said with confidence that the testing procedure outlined and employed in this study can be used to determine the impact of various design features on the drag coefficient, and thus the speed of the shell.
